# Use of Hepatitis C Viremic Donors to Expand the Pediatric Donor Pool

**DOI:** 10.3390/pathogens13110947

**Published:** 2024-10-30

**Authors:** Natasha Dilwali, Christopher Hartley, Paul K. Sue, Wikrom Karnsakul

**Affiliations:** 1 Division of Pediatric Gastroenterology, Hepatology and Nutrition, Department of Pediatrics, Johns Hopkins University of Medicine, Baltimore, MD 21287, USA; wkarnsa1@jhmi.edu; 2 Department of Pharmacy, The Johns Hopkins Hospital, Baltimore, MD 21287, USA; chartle8@jh.edu; 3 Division of Pediatric Infectious Diseases, Department of Pediatrics, Columbia University Irving Medical Center, New York, NY 10032, USA; pks2152@cumc.columbia.edu

**Keywords:** hepatitis C virus (HCV), direct-acting antiviral (DAA) therapies, pediatric organ transplant, HCV-positive donors

## Abstract

The use of hepatitis C virus (HCV)-positive donors in organ transplantation has become increasingly viable due to advancements in direct-acting antiviral (DAA) therapies, which offer high cure rates. This review aims to evaluate the current practices, benefits, and challenges of utilizing HCV-positive donors for organ transplantation. The recent data show that transplant centers are progressively accepting HCV-positive donors for various organs, including kidneys, livers, and hearts, given the efficacy of post-transplant antiviral treatment. Using these donors has helped mitigate the organ shortage crisis, increasing the donor pool and reducing waitlist times. Despite these advantages, the approach raises concerns about viral transmission, long-term outcomes, and the cost-effectiveness of post-transplant DAA therapy. Furthermore, this review highlights the ethical implications of informed consent and the monitoring of HCV-negative recipients following transplantation. The outcomes from recent studies suggest that with proper management, transplantations from HCV-positive donors to HCV-negative recipients can be safe and effective, leading to excellent graft function and patient survival. This review synthesizes existing research and offers insights into optimizing protocols for future transplants involving HCV-positive donors.

## 1. Introduction

The demand for pediatric liver transplants continues to exceed the supply of available donors, leading to prolonged wait times and increased morbidity and mortality. In the 2021 Annual Organ Procurement and Transplantation Network (OPTN)/Scientific Registry of Transplant Recipients (SRTR) Data Report, 36 pediatric patients died or were removed for being too sick to undergo a transplant on the waitlist. In addition, 666 new pediatric patients were added to the waitlist and almost a third of candidates waited two or more years until transplant, making it evident that new strategies are needed to expand the donor pool. Despite the clear need, the proportion of children undergoing living donor transplants has not changed significantly in the past decade [1]. 

One potential solution is the use of hepatitis C virus (HCV) viremic organs for pediatric transplantation. The advent of direct-acting antivirals (DAAs) has revolutionized the treatment of HCV, with the Food and Drug Administration (FDA) approving the first agents for adults in 2011 and children in 2017 [2,3]. These highly effective medications, now approved for use in children as young as three years, have demonstrated a sustained virologic response (SVR) greater than 90% in most HCV genotypes [3]. This development has led to the increased utilization of HCV-viremic organs in adult aviremic transplants, with studies showing comparable early graft outcomes to nonviremic donors [4,5,6,7]. 

Given the high level of safety and efficacy of DAAs compared to prior interferon-based regimens, the underutilization of HCV-viremic donors in pediatric liver transplants is concerning [6]. OPTN data reveals that HCV-viremic livers were three times more likely to be discarded compared to HCV-negative livers from 2005 to 2010, with the discard rate decreasing to 1.7 times after 2013. Moreover, these organs often come from younger, more optimal donors, particularly in the context of the opioid epidemic [2]. 

For pediatric patients facing clinical deterioration or whose Pediatric End-Stage Liver Disease (PELD) scores do not accurately reflect their degree of illness, the benefits of accepting an HCV-viremic organ may outweigh the risks. The PELD model is a formula used to assign priority on the waitlist to transplant candidates younger than 12 years of age based on medical urgency. It is crucial to consider these organs as viable options to improve survival rates, quality of life, and reduce healthcare costs and waitlist times.

It is important to note that the perspective presented in this review is primarily North American, specifically based on the healthcare system and treatment strategies in the United States (US). In the US, access to DAAs, organ donation systems, and financial frameworks, such as insurance coverage and healthcare policies, directly influence the utilization of HCV-viremic organs. These factors may differ significantly in other regions, where variations in the treatment protocols, pharmaceutical approvals, and organ allocation systems exist. While the underlying biology and efficacy of DAAs may be global, the practical application of these strategies is influenced by local healthcare contexts.

## 2. Disease Background

### 2.1. Prevalence

The global reach of HCV infection in children and adolescents is estimated to affect approximately five million individuals. These reports likely underestimate the true burden due to inconsistent screening practices globally. Limited access to diagnostic tools and variability in the healthcare infrastructure contribute to these discrepancies. In the US, prevalence rates in the pediatric population range from 0.05% to 0.36% and are rising [3]. This increase is partially attributed to factors such as maternal transmission and the opioid crisis. Additionally, it is believed that only 20% of individuals are aware of their diagnosis, highlighting the need for broader awareness and improved screening efforts [8]. 

### 2.2. Infection

HCV is classified into eight genotypes, with genotypes 1 and 2 being the most common in Western countries, including the US, Genotype 3 is more prevalent in South and East Asia but is also seen in the US. In contrast, genotypes 4 through 8 are primarily found in Africa, Asia, and the Middle East. Despite the regional variations, the genotype distribution among children generally mirrors that of adults [3,9]. Acute HCV infection in children is rare, and most pediatric cases involve chronic infection, often with minimal or mild symptoms. Approximately 25% to 40% of children with HCV will experience spontaneous clearance of viremia by two years of age, with an additional 6% to 12% clearing the infection by adulthood [3,10].

However, fibrosis can still develop in some children, though advanced liver disease is uncommon before adulthood. Understanding the nuances of acute HCV infection in children is particularly important when considering the use of viremic organs in transplantation, as acute infection in transplant recipients could complicate outcomes [3,11].

### 2.3. Diagnosis

HCV infection is typically diagnosed through serologic tests, such as enzyme immunoassays and chemiluminescence assays, which detect antibodies within two-to-six months after exposure. Nucleic acid testing (NAT) is also used to detect viral RNA directly. Both serology and NAT are essential in assessing donor status, particularly in transplantation settings. In post-transplant recipients, NAT is preferred as serology often fails to detect early donor-transmitted HCV or acute infection during the antibody window period. The risk of transmission varies depending on both antibody testing and NAT status, with NAT-positive donors posing a higher transmission risk. While an HCV-positive antibody and NAT indicate an active infection with high transmission risk, HCV antibody testing alone cannot determine transmission. Ensuring that NAT testing is performed promptly is critical for appropriate management in transplant settings [2]. 

### 2.4. Transmission

Mother-to-child transmission, both in utero and at the time of delivery, remains the most common mode of infection in children. Approximately 31% of these transmissions occur in utero while 50–80% occur during the peripartum period [12]. Despite the risk of transmission, universal screening for HCV is not routinely performed in pregnant women and is reserved for those who are deemed high-risk. However, the American Association for the Study of Liver Diseases (AASLD) and the Infectious Diseases Society of America (IDSA) now recommend that all pregnant women be tested for HCV to better identify and manage potential cases [13]. 

In addition to perinatal transmission, HCV infection in adolescents and young adults is increasingly linked to intravenous drug use (IVDU), which has surged by 364% in this age group over the past decade. Other less common transmission routes include, but are not limited to, tattoos and piercings in unregulated settings, and sexual practices involving multiple partners or trauma [3]. 

## 3. Treatment

### 3.1. Interferon and Ribavirin, Why It Did Not Work

Before the introduction of DAAs in 2017, the standard treatment for HCV involved a combination of pegylated interferon (PEG-IFN) and ribavirin (RBV). This regimen was associated with significant challenges, primarily due to its severe and often debilitating side-effects which often outweighed the slow progression of the disease. Side-effects included bone marrow suppression, hemolytic anemia, thrombocytopenia, neuropsychiatric symptoms, ocular complications, and developmental blunting. Along with these more serious effects, patients also experienced more common side effects including fever, anorexia, gastrointestinal symptoms, and rashes [14]. These complications made the treatment poorly tolerated, particularly in the post-transplant population, leading to high recurrence rates of HCV and reduced survival outcomes [15]. 

### 3.2. Current Regimens for Pediatric Patients

The advent of DAA therapy has revolutionized HCV treatment, offering a simpler, shorter, and significantly more effective alternative to interferon-based regimens (Table 1) [14]. The primary goals of DAA treatment are to eradicate the virus, prevent transmission, and mitigate the long-term effects of chronic liver inflammation and injury. Clinical trials have demonstrated that DAA therapy achieves a sustained virologic response (SVR) rate of 96% just 12 weeks post-treatment, with an adverse effect profile comparable to the placebo. This high level of efficacy and safety has led to recommendations that DAA therapy be initiated in all children diagnosed with chronic HCV, regardless of the serum aminotransferase levels, to prevent the progression of liver disease, and address concerns such as fibrosing cholestatic hepatitis [13].

### 3.3. Which Regimen to Choose

When selecting a DAA regimen for pediatric patients, several key factors should be considered. Age is a critical determinant as DAAs are generally recommended for children who are at least three years old. The patient’s HCV genotype is another essential factor, often required by insurance companies before initiating treatment, as it influences the choice of the most effective antiviral agent. Additionally, the patient’s treatment history—whether they are treatment-naïve—and the status of their liver disease (compensated or decompensated) play significant roles in determining the appropriate regimen. These factors collectively guide clinicians in selecting the most suitable and effective treatment for each patient [16]. 

While the emergence of HCV resistance-associated substitutions (RAS) presents a potential challenge to the efficacy of DAA’s in the treatment of donor-derived HCV, studies to date have demonstrated a high barrier to resistance and high levels of efficacy with current DAA regimens [17]. As such, the routine testing of donors for resistance is generally not recommended, although in the setting of DAA experienced donors, or less common HCV genotypes (eg Genotype 3), virological failure secondary to RAS has been observed. As the pool of pediatric patients receiving viremic or treatment experienced donors expands, further research is necessary to help delineate what role, if any, RAS testing will play in improving post-transplant outcomes [18].

### 3.4. Post-Transplant When to Start Therapy and What to Monitor

The Infectious Diseases Society of America (IDSA) and American Association for the Study of Liver Diseases (AASLD) Hepatitis C Guidance 2023 panel recommends initiating DAA therapy within one to two weeks post-transplant, ideally within one week if the patient is clinically stable [16]. The early initiation of therapy with close monitoring is crucial to prevent the progression of HCV-related liver damage. In the THINKER trial, viremia (HCV RNA levels) and liver enzymes were monitored before treatment, at weeks 4, 8, and 12 during treatment, and then at 4 and 12 weeks post-treatment. Comprehensive metabolic panels (CMPs) were also conducted weekly for three to six months post-transplant [19]. The MYTHIC trial followed a different protocol, starting treatment as early as 2–3 days post-transplant, with viremia monitored at initiation, week 1, 4, and 8, and extending to weeks 8, 12, and 24 post-treatment [20]. Delays in initiating therapy, often due to insurance authorization challenges, can significantly hinder disease management. For instance, a study of 34 HCV-seronegative liver transplant recipients showed a median time to treatment of 27.5 days due to insurance difficulties, leaving patients untreated for nearly a month and at risk for disease progression [21]. 

### 3.5. Dosing and Interactions in Common Immunosuppressants in SOT

When considering the use of HCV-viremic donors, it is essential to evaluate potential interactions between DAAs and commonly used immunosuppressants in pediatric transplantation (Table 2). Typically, pediatric patients are managed with a regimen that includes steroids and tacrolimus, followed by a combination of tacrolimus, steroids, and a mycophenolate agent [1]. While most DAAs do not exhibit significant interactions with these immunosuppressants, some can affect the maximum concentration (Cmax) and area under the curve (AUC) of these drugs, necessitating dose adjustments to mitigate the risk of liver injury [15]. Although the adult literature has not shown significant graft dysfunction or rejection associated with these interactions, careful monitoring and dose modifications are essential to optimize the outcomes of both therapies [5]. Frequent monitoring is crucial to identify any potential interactions early and to ensure the safe and effective use of these medications in post-transplant care.

## 4. Experiences in Transplantation

### 4.1. Adult Experiences

In the adult transplant population, the scarcity of organs, combined with the increasing demand, has led to prolonged waitlist times and a need for innovative strategies to expand the donor pool. Institutions have responded by considering options such as increasing donations after circulatory death and accepting marginal or high-risk donors, including those with HCV viremia. DAA therapy has significantly contributed to this shift, as it allows for the effective treatment of HCV in recipients, thereby increasing the acceptance of HCV viremic organs by both institutions and aviremic recipients (Table 3).

Nucleic acid amplification testing (NAT) now enables the real-time identification of viremic donors, enhancing the safety and efficiency of using these organs. A notable study by Kapila et al. in 2020 documented the largest real-world experience of transplanting HCV viremic organs into aviremic recipients. The study included adult patients who had not undergone prior transplants, had a Model for End-Stage Liver Disease (MELD) score greater than 35 (score utilized for allocation of donors for liver transplantation with greater liver disease severity correlating to a higher MELD score), and were not pregnant. Patients were thoroughly informed of the risks and benefits and institutions committed to covering DAA therapy costs if insurance coverage was denied. Post-transplant, patients were closely monitored with HCV NAT and genotype testing to guide the initiation of DAA therapy. Treatment began once the patient was stabilized on immunosuppressive therapy and confirmed HCV NAT-positive. The study demonstrated that the use of DAAs was well-tolerated, with a high rate of a sustained virologic response (SVR) across multiple organ types [6].

Similarly, a study by Cotter et al. examined trends in organ utilization and graft survival using SRTR data. The analysis showed that in the post-DAA era, viremic organs accounted for 7.1% of all liver transplants, up from 3.7% in the pre-DAA era. The study also found improved graft survival rates, reduced waitlist times, and comparable early graft outcomes (2 years) between recipients of HCV-positive and HCV-negative organs, further supporting the use of viremic organs [4].

### 4.2. Pediatric Experiences

In the pediatric population, the exploration of high-risk donors, including those with HCV viremia, is being pursued as a means to reduce waitlist mortality and increase organ access. Recent reports in pediatric cardiology and pulmonology have demonstrated the feasibility of using HCV viremic organs in pediatric transplants (Table 3).

In 2022, the first case of a pediatric bilateral lobar lung transplant from an HCV viremic donor was reported. The recipient, who was acutely ill, received a transplant from a viremic donor who had sustained brain damage due to an overdose. DAA therapy was initiated six hours before the operation and continued for seven days postoperatively. At five months post-transplant, no viremia was detected, highlighting the protocol’s safety and efficacy in pediatric solid organ transplantation [23].

Earlier, in 2019, Yasmin Radzi et al. explored the use of hepatitis B core antibody-positive or HCV seropositive donors for pediatric heart transplantation. The study found that graft survival rates were comparable to those in recipients of aviremic donor organs. Additionally, the study noted that from 2008 to 2015, over 2200 potential high-risk donors were not utilized for pediatric thoracic transplantation, highlighting the potential to significantly expand the donor pool with these organs [24]. While these initial successes are promising, further studies with larger cohorts and standardized protocols are needed to solidify the use of HCV viremic organs in pediatric transplantation.

## 5. Moving Forward

The use of HCV viremic organs in transplantation offers substantial potential for both adult and pediatric populations, despite distinct differences in the underlying indications for transplant. While HCV is a common indication for liver transplantation in adults, pediatric transplants are driven by more heterogeneous causes, such as biliary atresia and metabolic liver diseases. This variability impacts the comparative use of HCV-positive donors. The growing availability of DAAs now allows the safe use of HCV-viremic donors, even in pediatric patients without a pre-existing HCV infection. For children facing urgent transplantation needs, accepting HCV-positive organs may help reduce waitlist mortality, making it a valuable strategy.

However, the implementation of safe protocols remains critical to prevent complications such as fibrosing cholestatic hepatitis. Equally important is the establishment of post-transplant monitoring protocols. A multidisciplinary approach involving surgeons, hepatologists, infectious disease specialists, and specialty pharmacies is essential for optimizing patient outcomes. The adult guidelines developed by Stewart et al. in 2020 emphasize patient education, informed consent, and insurance verification prior to transplantation, with recommended DAA treatment durations of 8 to 12 weeks, though shorter regimens have also been successful. Given the ongoing research into long-term outcomes, institutions need to engage their Institutional Review Boards (IRBs) when implementing programs that utilize viremic organs [7].

Despite the positive outcomes seen in trials like THINKER, EXPANDER, and MYTHIC, where all patients achieved a sustained virologic response post-transplantation, challenges persist, especially ensuring early and uninterrupted access to DAA therapy [20,25,26]. A study found that while all patients eventually received insurance coverage for DAA therapy, 95% required prior authorization, and 24% had to appeal to obtain coverage. Additionally, patients with Medicaid experienced significantly longer approval times (31.5 vs. 6 days) [21]. This highlights the need for transplant centers to support their patients in navigating these barriers, possibly by covering the cost of DAA therapy when insurance issues arise.

For pediatric patients, these challenges are heightened by the scarcity of acute HCV infection data, making the timing and efficacy of treatment less certain. Clinical trials are urgently needed to assess drug efficacy, toxicities, and long-term outcomes. Additionally, the low awareness and acceptance of HCV viremic organs among transplant candidates highlight the need for effective patient education to alleviate stigma and ensure informed decision making [3]. A study involving 50 adult patients awaiting transplants revealed that only 60% were aware that HCV is a curable disease, and just 46% were willing to accept an HCV viremic organ. This emphasizes the critical need for medical teams to provide accurate, up-to-date information about HCV infections and the use of viremic organs to alleviate fears and misconceptions [27].

Overall, the use of HCV viremic organs is a promising strategy to reduce waitlist times and expand the donor pool. The encouraging results seen in adult populations suggest that this approach could be feasible in pediatrics, particularly for critically ill patients. However, the success of this strategy will depend on the continued development of protocols, patient education, and research to ensure safety and efficacy across all populations.

## Figures and Tables

**Table 1:** Current FDA-Approved Regimens for Treatment-Naïve Hepatitis.

Medication	Approval	Duration	SVR12	Genotypes
Sofosbuvir + Ledipasvir (Harvoni^®^)	2017 for ≥12 yo 2019 for ≥3 yo	12 weeks	98%	1,4,5,6
Sofosbuvir + Ribavirin	2017 for ≥12 yo 2019 for ≥3 yo	12 weeks	98%	2,3
Sofosbuvir + Velpatasvir (Epclusa^®^)	2020 for ≥12 yo 2021 for ≥3 yo	12 weeks	98%	1,2,3,4,5,6
Glecaprevir + Pibrentasvir (Mavyret^®^)	2019 ≥12 yo 2021 for ≥3 yo	12 weeks	96%	1,2,3,4,5,6

**Table 2:** Pharmacokinetics of Approved Hepatitis C Medications commonly used in the USA [22].

Medication Class	Mechanism of Action	Generic Medication Name	Pharmacokinetics (Studied from Adult Data)
Interferon-Based Therapies	Initiate intracellular activity via modulation of cell cycle to cause cytotoxic effects to lymphocytes and virus-infected cells	Interferon-Alpha	A: N/A (Intramuscular/Intravenous) D: Large volume of distribution M/E: Renal metabolism, elimination half-life of approximately 2 h
	Works against RNA viruses by inhibiting HCV polymerase, but mechanistically not fully understood	Ribavirin	A: Bioavailability increased with high-fat meals D: Large volume of distribution M/E: Renal/hepatic metabolism (not CYP450 enzymes)
		Ledipasvir	A: Not changed by food D: >99.8% protein-bound M/E: CYP1A2, CYP2C8, CYP2C9, CYP2C19, CYP2D6, and CYP3A4; fecal elimination, 70% unchanged in the feces
		Pibrentasvir	A: Increased by food D: >99.9% protein-bound M/E: No metabolism, fecal elimination
		Velpatasvir	A: Increased by food D: >99% protein-bound M/E: CYP2B6/2C8/3A4 metabolism, biliary excretion
		Sofosbuvir	A: Increased by food D: 61–65% protein-bound M/E: Hepatic metabolism (not CYP), Glomerular filtration, and active tubular secretion elimination
		Glecaprevir	A: Increased by food D: 97.5% protein-bound M/E: Secondary metabolism via CYP3A4, fecal elimination
		Voxilaprevir	A: Increased by food D: >99% protein-bound M/E: CYP3A4 metabolism, biliary excretion

Legend: A, absorption; D, distribution; M/E, metabolism/elimination; N/A, not applicable. Used with permission.

**Table 3:** Solid HCV Viremic Organs Transplanted to Aviremic Recipients.

	Solid HCV Viremic Organs Transplanted to Aviremic Recipients
Adult	Kidney, Liver, Pancreas, Heart, Lung
Pediatric	Lungs [23], Heart [24], Kidney [24]

## Data Availability

No new data were created or analyzed in this study. Data sharing is not applicable to this article.
